# How Can a Ketogenic Diet Improve Motor Function?

**DOI:** 10.3389/fnmol.2018.00015

**Published:** 2018-01-26

**Authors:** Charlotte Veyrat-Durebex, Pascal Reynier, Vincent Procaccio, Rudolf Hergesheimer, Philippe Corcia, Christian R. Andres, Hélène Blasco

**Affiliations:** ^1^Département de Biochimie et Génétique, Centre Hospitalier Universitaire, Angers, France; ^2^INSERM 1083, CNRS, Equipe Mitolab, Institut MITOVASC, UMR 6015, Université d’Angers, Angers, France; ^3^INSERM U930, Université François Rabelais de Tours, Tours, France; ^4^Service de Neurologie, Centre Hospitalier Universitaire de Tours, Tours, France; ^5^Laboratoire de Biochimie et Biologie Moléculaire, Centre Hospitalier Universitaire de Tours, Tours, France

**Keywords:** ketogenic diet, motor function, motor neuron, β-hydroxybutyrate, ketone bodies, neuromuscular diseases

## Abstract

A ketogenic diet (KD) is a normocaloric diet composed by high fat (80–90%), low carbohydrate, and low protein consumption that induces fasting-like effects. KD increases ketone body (KBs) production and its concentration in the blood, providing the brain an alternative energy supply that enhances oxidative mitochondrial metabolism. In addition to its profound impact on neuro-metabolism and bioenergetics, the neuroprotective effect of specific polyunsaturated fatty acids and KBs involves pleiotropic mechanisms, such as the modulation of neuronal membrane excitability, inflammation, or reactive oxygen species production. KD is a therapy that has been used for almost a century to treat medically intractable epilepsy and has been increasingly explored in a number of neurological diseases. Motor function has also been shown to be improved by KD and/or medium-chain triglyceride diets in rodent models of Alzheimer’s disease, Parkinson’s disease, amyotrophic lateral sclerosis, and spinal cord injury. These studies have proposed that KD may induce a modification in synaptic morphology and function, involving ionic channels, glutamatergic transmission, or synaptic vesicular cycling machinery. However, little is understood about the molecular mechanisms underlying the impact of KD on motor function and the perspectives of its use to acquire the neuromuscular effects. The aim of this review is to explore the conditions through which KD might improve motor function. First, we will describe the main consequences of KD exposure in tissues involved in motor function. Second, we will report and discuss the relevance of KD in pre-clinical and clinical trials in the major diseases presenting motor dysfunction.

## Introduction

The KD, tested for the first time in 1921 for intractable childhood epilepsy, is based on a normocaloric, high fat, adequate-protein, and low-carbohydrate diet resulting in the production of KBs (Keene, 2006). Different types of KDs have been described. The classic ketogenic therapy is based on a diet providing 90% of calories from long-chain fatty acids, a restricted protein portion (1 g/kg/day), and minimal carbohydrates. Traditionally, the diet is comprised of four parts fat, mainly LCTs, for one part carbohydrates and proteins. The ratio can be modified to 3:1, 2:1, or 1:1, respectively, similar to the modified Atkins diet (Kossoff et al., 2003). The MCTs diet is also proposed with 60% of calories from octanoate and decanoate that are more ketogenic than LCTs (Huttenlocher, 1976). The last alternative to a ketogenic therapy is the low glycemic index diet characterized by higher amounts of carbohydrates with low glycemic index (Coppola et al., 2011).

Despite the underlying, unclear mechanisms, KD is considered to be a “neuroketotherapeutic” (Koppel and Swerdlow, 2017). The efficacy of KD in drug-resistant epilepsy in children and adult patients has been proven for almost a century (Stafstrom and Rho, 2012) with more than 50% reduction in seizures for intractable childhood epilepsy (Lefevre and Aronson, 2000). KD has progressively gained interest for the treatment of other diseases as a stand-alone metabolic therapy or as part of a general, therapeutic strategy (Paoli et al., 2014). Various mechanisms have been advocated to explain the anti-convulsive and neuroprotective effects of KD, such as a decrease in glucose metabolism due to the increase in lipid oxidation, a reduction in ROS production, an increase in ATP, and modulations of neuronal membrane excitability, inflammation, oxidative stress, and mitochondrial function (Gasior et al., 2006; Elia et al., 2017).

Thus, KD is expected to be highly relevant in diseases characterized by any of these mechanisms. For example, motor dysfunction, involving the nervous system, muscles and tendons, observed in neuromuscular diseases or as a component of various pathological conditions, may benefit from such treatment. As non-pharmacological management is rarely considered and little data has been published on dietary therapies, we have focused our review on the potential benefit of such KD therapies on motor function. Firstly, we will describe the neuroprotective effects of KD, especially in tissues involved in motor function. Secondly, we will present and discuss pre-clinical and clinical trials of KD for diseases presenting a motor dysfunction. Finally, we will present some perspectives of other new therapeutics, based on metabolic factors targeting energy metabolism.

## Protective Effects of KD on the Neuromuscular System

**FIGURE 1:**
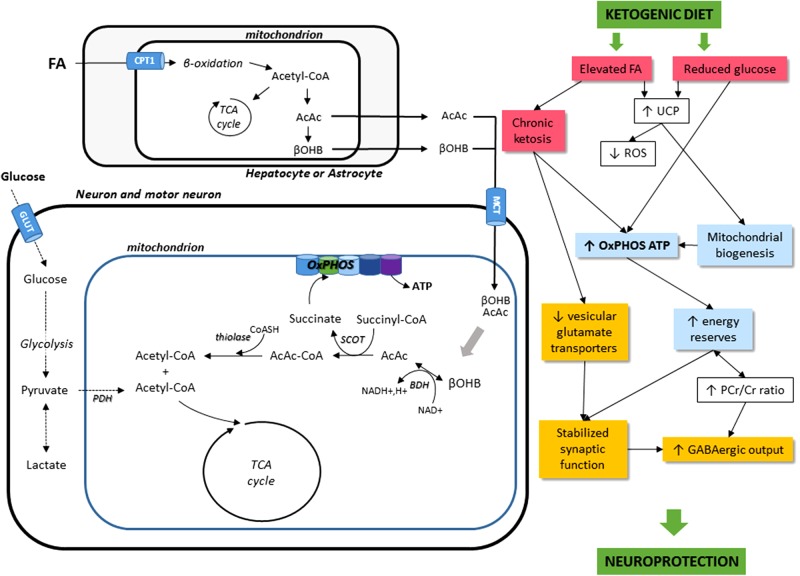
Schematic illustration of the main cellular mechanisms in the KD on the neuromuscular system. The main energetic substrate of the nervous system is glucose, but alternative substrates exist, for instance pyruvate (Gonzalez et al., 2005), lactate (Dienel, 2012), and βOHB (Kunnecke et al., 1993; Achanta and Rae, 2017). Most KBs (β-hydroxybutyrate and acetoacetate AcAc) used by the brain are supplied by the liver, but they can also be synthesized in astrocytes, which are the only cell type in the brain capable of oxidizing fatty acids (FA). KBs enter cells via the monocarboxylate transporter (MCT). Then, acetyl-CoA can enter the TCA cycle to produce ATP. KD leads to a decrease in glycolytic ATP generation and an increase in ATP generation in mitochondria. FA, fatty acids; CPT1, carnitine palmitoyltransferase 1; AcAc, acetoacetate; βOHB, β-hydroxybutyrate; OxPHOS, oxidative phosphorylation; TCA cycle, tricaboxylic acid cycle; ATP, adenosine triphosphate; BDH, beta-hydroxybutyrate dehydrogenase; SCOT, succinyl-CoA:3-ketoacid coenzyme A transferase; PDH, pyruvate dehydrogenase; GLUT, glucose transporters; UCP, uncoupling proteins; ROS, reactive oxygen species; PCr/Cr, phosphocreatine/creatine; GABA, gamma-aminobutyric acid.

The effects of KD administration on the neuromuscular system come through different mechanisms. For one, KD can directly induce metabolic shifts due to the high blood levels of KBs and to the restriction of carbohydrate intake (Danial et al., 2013). KD can also modify nutrient-integrating pathways, such as the mTOR pathway, involved in autophagy and mitophagy-related mitochondrial renewal. Finally, KD might have potential, indirect roles, such as effects on neurotransmission, oxidative stress, and inflammatory mechanisms. **Figure 1** sums up the cellular mechanisms induced by KD.

### Metabolism Switch

Ketogenic diet has a high impact in tissues with a high-energy requirement and with challenges from modifications in metabolic substrates, such as the neuromuscular system. The brain represents 2% of one’s body weight but consumes about 20% of the body’s energy stores (Belanger et al., 2011) in order to fuel the processes of neurotransmitter production/recycling, vesicular trafficking, maintenance of ion gradients for the propagation of action potentials, and memory, to name a few. Similarly, in the resting state, 20% of the energy expenditure is devoted to muscle (Gallagher et al., 1998), and it can largely increase with muscle contractions, for example for transforming organelles, enzymatic activities, intracellular signaling, and transcriptional responses (Coffey et al., 2007).

Ketogenic diet promotes KBs (acetoacetate and β-hydroxybutyrate) production in the liver from acetyl-CoA formed during mitochondrial β-oxidation of fatty acids. Some of the acetyl-CoA enters the TCA cycle, and the excess is used to form acetoacetate, which could be converted to βOHB by βOHB dehydrogenase (BDH) enzyme or spontaneously converted to acetone (Newman and Verdin, 2014). KBs are transported to other tissues (brain, muscle, and heart) through the blood and used as fuel, especially in the brain (Kunnecke et al., 1993; Rae et al., 2012; Achanta and Rae, 2017). It has also been reported that astrocytes can produce KBs from fatty acids (Auestad et al., 1991) and leucine (Bixel and Hamprecht, 1995). Astrocytes present the same preference for fatty acids (rather than glucose) as metabolic fuel and have enzymatic machinery similar to that of cultured hepatocytes (Guzman and Blazquez, 2001).

β-Hydroxybutyrate and acetoacetate enter cells via the MCT and provide an alternative substrate for brain. Several studies seem to highlight that KBs are a preferred carbon source under certain conditions (LaManna et al., 2009; Lund et al., 2011; Zhang Y. et al., 2013; Chowdhury et al., 2014; Valente-Silva et al., 2015), but these finding remain controversial (McKenna, 2012; Achanta et al., 2017). Some studies evoked that KBs provide more efficient energy source than glucose, even for the brain. They are metabolized faster than glucose and bypass the glycolytic pathway by directly entering the TCA cycle, whereas glucose has to first undergo glycolysis (Veech et al., 2001; LaManna et al., 2009; Elamin et al., 2017). Consequently, KBs lead to a decrease in glycolytic ATP generation and an increase in ATP generation by mitochondrial oxidation (Kim et al., 2010; Steriade et al., 2014; Hyatt et al., 2016). Moreover, Elamin et al. (2017) showed that whereas both glucose and KBs produce two molecules of acetyl-CoA, glucose reduces four molecules of NAD^+^ and KBs reduce either one (for conversion of BOHB in acetoacetate), or none (for conversion of acetoacetate in acetyl-CoA) during acetyl-CoA synthesis. KD is associated with a coordinated upregulation of hippocampal genes encoding metabolic and mitochondrial enzymes (Bough et al., 2006). KD also leads to fatty acid-mediated activation of peroxisome proliferator-activated receptor α (PPARα) that inhibits glycolysis and fatty acid synthesis, promotes the transcription of ketogenic enzymes, promoting mitochondrial and peroxisomal fatty acid oxidation (Cullingford, 2004). Moreover, the cellular energy reserve is increased as a result from a higher phosphocreatine/creatine ratio in the hippocampal tissue (Bough et al., 2006).

Taken altogether, neurons possess a better resistance and adaptive ability to metabolic stress and challenges, both having to do with a more energy-efficient fuel source and a larger mitochondrial load based on a stimulation of mitochondrial biogenesis (Bough et al., 2006; Srivastava et al., 2012, 2013).

### Antioxidant Effects

The accumulation of certain metabolites and hypoxia produced during muscular contractions, along with the high energetic requirement of the brain, might increase ROS production through the mitochondrial electron transport chain (Morales-Alamo and Calbet, 2014). Despite possible, positive effects of ROS production on mitochondrial adaptations (Ji et al., 2016), the deleterious effect of oxidative stress must be controlled, primarily in the brain (Islam, 2017). Veech (2014) reported a decrease in free radical production through the reduction of coenzyme Q, induced by KBs. Importantly, a decrease in oxidative stress and an increase in mitochondrial glutathione and glutathione peroxidase activity were observed during KD, which may protect tissues from injury (Ziegler et al., 2003; Bough et al., 2006; Stafford et al., 2010; Stafstrom and Rho, 2012; Greco et al., 2016). As ROS generation partially reflects mitochondrial function, the decrease in ROS production may be a result of an effect on NADH oxidation or on calcium overload (Kim et al., 2007; Maalouf et al., 2007). Some authors have described that, in KD, there is an elevated production of mitochondrial uncoupling proteins, thereby decreasing ROS levels probably via fatty acids found elevated in treated patients (Fraser et al., 2003; Sullivan et al., 2004; Maalouf et al., 2007). Carbohydrate restriction also induces stress response proteins, leading to a lowering of ROS and the maintenance of mitochondrial function (Lee et al., 1999). Tieu et al. (2003) observed a beneficial effect of KD in restoring mitochondrial Complex I function following inhibition by pharmacological agents. These results point out a central role of KD in oxidative stress management associated with the mitochondria.

### Synaptic Transmission

Numerous reports have suggested that the anticonvulsive mechanisms behind ketosis are based on a metabolic shift between the neurotransmitters GABA and glutamate, resulting in an increased inhibition and/or decreased excitation (Bough and Rho, 2007; Yudkoff et al., 2007). This could spark attention toward neuromuscular transmission (Diana et al., 2017). Indeed, KD can induce an increase in glutamate decarboxylase expression in the striatum of rats (Cheng et al., 2004) and an alteration of astrocytic GABA degradation via a modification of GABA transaminase activity (Suzuki et al., 2009). Moreover, GABA content is increased by KBs in rat brain synaptosomes (Erecinska et al., 1996), rat hippocampus (Calderon et al., 2017), and in the brain of patients (Wang et al., 2003). Some authors have reported the inhibition of glutamatergic, synaptic transmission (Danial et al., 2013; Lutas and Yellen, 2013), which implies a blockade of vesicular glutamate transporters (Juge et al., 2010). This interference with glutamate-mediated toxicity, implied in neuronal injury, could explain the interest for KD in neurological diseases.

### Signaling Pathways

Ketogenic diet could modulate crucial mechanisms in cellular homeostasis. For example, mTOR and AMPK pathways involved in cell proliferation, energetic metabolism, or protein biosynthesis could be implicated. KD induces the binding of insulin and free IGF-1 to their specific tyrosine kinase receptors and activates the phosphatidylinositol-3 kinase (PI3K)-Akt-mammalian target of rapamycin complex 1 (mTORC1). However, this effect is counteracted by the decrease in the intracellular ATP/AMP ratio and the activation of liver kinase B1 (LKB1)-AMP-activated protein kinase (AMPK) signaling, inhibiting mTORC1 (Newman and Verdin, 2014). Such effects may be essential for motor dysfunction in ALS, for example, as deregulation in mTOR and AMPK signaling pathways has been described in this disease (Saxena et al., 2013; Perera and Turner, 2016).

### Anti-inflammatory Effects

Fasting and KD have been associated with effects on inflammatory mechanisms (Stamp et al., 2005; Dupuis et al., 2015). Some authors have observed an elevated expression of cytokine interferon-γ in the hippocampus of rats, thus protecting cells from excitotoxicity (Lee et al., 2006). Moreover, fatty acids activate PPARα which, in turn, inhibits the pro-inflammatory NF-κB and cytokines, such as IL-6 and TNFα (Cullingford, 2004; Nandivada et al., 2016). βOHB inhibits the NLRP3 inflammasome, which controls the activation of caspase-1 and the release of the pro-inflammatory cytokines, IL-1β and IL-18 (Youm et al., 2015; Goldberg et al., 2017).

## Use of KD in Motor Dysfunction

The beneficial effect of KD has been hypothesized in various diseases, such as epilepsy, metabolic defects, cancers, autism, depression, migraines, narcolepsy, Parkinson’s disease, and Alzheimer’s disease (Vanitallie et al., 2005; Baranano and Hartman, 2008; Newport et al., 2015). Also, a therapeutic effect has been proposed for diseases with substantial motor dysfunction, as reported in **Table 1** summing the main pre-clinical and clinical evaluations of KD in these diseases.

**Table 1:** Main preclinical and clinical evaluations of KD and treatments derived from the KD in diseases with motor dysfunction.

Disease	Pre-clinical evaluation			Clinical evaluation		
	Type of study	Main findings	Reference	Type of study	Main findings	Reference
Amyotrophic lateral sclerosis (ALS)	KD, MCT, or DP in SOD1-G93A transgenic ALS mouse model	Longer maintenance of motor function Decrease in motor neuron death Delay onset of motor symptoms	Zhao et al., 2006, 2012; Ari et al., 2014; Tefera et al., 2016	Clinical trial of KD use, phase III NCT01016522	No result provided	Not published
Angelman syndrome (AS)	KE-treated AS mouse model	Improvement of motor coordination No effect on locomotor activity	Ciarlone et al., 2016	Case reports (KD and low glycemic index diet in AS patients)	Decrease in seizures	Evangeliou et al., 2010; Thibert et al., 2012
Mitochondrial myopathy	KD in mouse model	Delayed disease progression	Ahola-Erkkila et al., 2010	Pilot study KD in patients	Improvement of muscle strength and delayed disease progression	Ahola et al., 2016
Alzheimer’s disease	KD in APP/PS1 and Tg4510 mouse models KE in Alzheimer mouse models	Improvement of motor function Improvement in energy metabolism and reduction in amyloid deposition	Beckett et al., 2013; Brownlow et al., 2013 Zhang J. et al., 2013; Pawlosky et al., 2017	Pilot study for assessment of MCT tolerance in Alzheimer patients KE in a case of Alzheimer’s disease	Good tolerance No improvement in cognitive function No assessment of motor function Gain in cognitive function and daily motor activity	Ohnuma et al., 2016 Newport et al., 2015
Spinal cord injury (SCI)	KD in rats with SCI	Improvement of functional forelimb	Streijger et al., 2013	Clinical trial of KD use	Safety and feasibility in patients with acute SCI	Guo et al., 2014
Parkinson’s disease	KD or βOHB in rodents models	Protection of dopaminergic neurons from degeneration Improvement of motor function	Tieu et al., 2003; Cheng et al., 2009; Yang and Cheng, 2010; Shaafi et al., 2016	Feasibility study of KD use	Improvement of unified Parkinson’s disease rating scale score, including motor function	Vanitallie et al., 2005
Rett syndrome	Restricted KD or MCT in Rett syndrome (Mecp2 KO) mice	Improvement of motor behavior and reduction in anxiety Augmented survival, improvement in mitochondrial morphology	Mantis et al., 2009; Park et al., 2014	Cases reports (KD)	Improvement of motor function	Haas et al., 1986; Liebhaber et al., 2003
GLUT 1 deficiency				Case report KD use in GLUT1 patients Evaluation of KD short effect in GLUT1 patients	Improvement of motor function Mainly improvement of cognitive function, with improvement of language and physical endurance Moderate improvement of motor disorder	Friedman et al., 2006 Ramm-Pettersen et al., 2014 Bertoli et al., 2015

*ALS, amyotrophic lateral sclerosis; AS, Angelman syndrome; βOHB, β-hydroxybutyrate; DP, Deanna protocol; KD, ketogenic diet; KEs, ketone esters; MCTs, medium-chain triglycerides.*

### Amyotrophic Lateral Sclerosis (ALS)

#### Rational of the Use of KD in ALS

Amyotrophic lateral sclerosis is a fatal, neurodegenerative condition characterized by motor neuron degeneration that leads to progressive motor weakness and death between 2 and 5 years from onset (Korner et al., 2013). Many molecular mechanisms have been involved in ALS pathophysiology, but the starting point of the disease remains unclear. Drug development has yielded few successes, and the prognosis has not changed dramatically since the first reports, nearly 150 years ago.

Regarding studies on ALS mouse models, Siva (2006) proposed KD as a promising strategy to slow down the progression of ALS, especially through the increase in mitochondrial function. The main mechanisms related to mitochondrial malfunction and ALS have been recently reviewed (Carri et al., 2017).

The heterogeneous effects of KD on the neuromuscular system could support the interest in KD treatment in a heterogeneous disease, such as ALS. In fact, many hypotheses have been raised on ALS pathophysiology. Among the known gene mutations associated with ALS, *TAR DNA-binding protein (TARDPB)* gene is related with neuronal density of mitochondria and cristae formation (Xu et al., 2010; Stribl et al., 2014), *SOD1* is associated with oxidative stress, and *CHCHD10* is involved in oxidative phosphorylation and the maintenance of mitochondrial cristae morphology (Bannwarth et al., 2014). Other authors have described a decreased activity in mitochondrial Complex I in skeletal muscle and the spinal cord of ALS patients (Vielhaber et al., 2000). Moreover, several reports have highlighted metabolic alterations in this disease, enabling a possible role of therapeutics targeting energetic metabolism (Dupuis et al., 2008; Blasco et al., 2016). Various studies have directly observed shifts in TCA cycle intermediates, such as decreased C4-intermediate levels in ALS mice (Niessen et al., 2007), or decreased oxidative phosphorylation and ATP production in cellular models, transgenic mice and ALS patients (Jung et al., 2002; Mattiazzi et al., 2002; Menzies et al., 2002; Wiedemann et al., 2002; Browne et al., 2006). GABA imbalance and glutamate neurotransmission are also involved in ALS pathophysiology and may be relevant to modulate (Blasco et al., 2014; Diana et al., 2017). Likewise, the roles of neuro-inflammation and oxidative stress have often been indicated in *in vitro* and *in vivo* experiments, justifying a therapeutic approach also focused on these mechanisms (Liu and Wang, 2017; Liu et al., 2017). All these mechanisms involved in ALS pathophysiology could justify the use of KD as therapy in this disease.

#### Evaluation of KD in ALS

Zhao et al. (2006) reported in a study that a higher motor neuron survival and an improved motor function resulted from KD administration through the gain in mitochondrial energy production in SOD1-G93A transgenic ALS mice. They noticed that KD-fed mice maintained motor function longer than SOD1-mutant mice fed a SD and had more intact motor neurons in the lumbar spinal cord. They also demonstrated that βOHB prevented rotenone-mediated inhibition of mitochondrial Complex I (Zhao et al., 2006). Zhao et al. (2012) confirmed the promising therapeutic approach by way of improvement in mitochondrial function and motor neuron count in an ALS mouse model following administration of caprylic acid, a MCT precursor of KB.

The link between lipid concentrations and survival remains an enigma in ALS. Nevertheless, a recent study has revealed that higher caloric intake improves survival and that low cholesterol may end up being deleterious in ALS patients (Blasco et al., 2017). A phase III clinical trial evaluating the safety and the tolerance of KD in ALS patients fed through a gastrostomy tube has been conducted in the United States (clinicaltrials.gov NCT01016522), but results have not yet been published. The primary outcome measure is to evaluate the prevention of malnutrition in ALS patients but not to identify the benefit in the disease’s prognosis.

### Angelman Syndrome (AS)

#### Rational of the Use of KD in AS

Angelman syndrome is a devastating, neurological disorder with no treatment. AS patients suffer from motor dysfunction, intellectual disability, frequent smiling and laughter, lack of speech, and severe seizures. This syndrome is due to an alteration in the E3 ubiquitin ligase (Margolis et al., 2015). The main pathophysiological pathways concern an impairment in synaptic plasticity, deregulated dopaminergic and GABAergic neurons, abnormal mTOR signaling in the cerebellum, abnormal cell contact signaling and excitation/inhibition imbalance (Bi et al., 2016). It has been shown, as well, that the stimulation of mitochondrial biogenesis by KD improves the hippocampal deficits in AS mice (Bough et al., 2006; Su et al., 2011).

#### Evaluation of KD in AS

Ciarlone et al. (2016) studied the effect of KEs, precursors of KBs, in AS mouse models. They investigated behavioral and metabolic outcomes, as well as cognitive and motor functions in AS and control mice either receiving KEs or not.

The major findings highlighted that KEs supplementation improves motor coordination but does not affect general locomotor activity in AS mice. The average latency for falling on the accelerating rotarod was significantly increased in AS mice treated with KE compared to untreated AS mice, but the performances did not reach those of the control mice. KE-treated AS mice showed a significant increase in the latency on the wire hang test compared to AS mice, but the latency remained inferior to WT mice for which KE had no effect. The severity of the hindlimb clasping score was significantly decreased in KE-treated AS mice compared to untreated AS mice.

Both the KD and the low glycemic index diets have been administered to patients and have illustrated promising results in seizures of AS (Evangeliou et al., 2010; Thibert et al., 2012), suggesting that the benefit rather stems from carbohydrate restriction and not from increasing blood KB levels (Gano et al., 2014).

### Mitochondrial Myopathy

Mitochondrial disorders are clinically and genetically heterogeneous diseases with a neuromuscular component caused by mutations either in mitochondrial DNA or in nuclear genes encoding mitochondrial proteins. Interestingly, KD has showed a relevant effect on a mouse model for late-onset mitochondrial myopathy characterized by generalized muscle weakness (Ahola-Erkkila et al., 2010). KD decreased the amount of Cytochrome c Oxidase-negative muscle fibers and protected mitochondrial ultrastructure in the muscle. A recent study suggested that the use of KD in mitochondrial myopathy enhanced muscle strength and delayed the progression of the disease but might induce muscle damage in a subpopulation (Ahola et al., 2016).

### Other Diseases with a Neuromuscular Component

Various studies have supported a regeneration in motor performance with KD in rodent models of Alzheimer’s disease, spinal cord injury, Parkinson’s disease, and Rett syndrome. Various mouse models of Alzheimer’s disease (i.e., mice carrying mutations in amyloid precursor peptide, APP, and/or presenilin, PS, as models of amyloid deposition, and Tg4510 mouse model as a model of tau deposition) have presented an improved latency for falling on Rotarod apparatus under KD without a reduction in β-amyloid or tau accumulation (Beckett et al., 2013; Brownlow et al., 2013). KD improved forelimb motor function in rodents after spinal cord injury with maintenance of the functional benefits when returning to a SD after 12 weeks of KD (Streijger et al., 2013). A number of studies have suggested that KD and βOHB administration protect dopaminergic neurons from degeneration (Kashiwaya et al., 2000) and improve motor function in rodent models of Parkinson’s disease (Tieu et al., 2003; Cheng et al., 2009; Yang and Cheng, 2010; Shaafi et al., 2016). Mantis et al. (2009) illustrated a recovery in motor behavior in Rett syndrome mice, principally by caloric restriction in the diet.

The use of KD in these diseases presenting a neuromuscular component was also studied in human patients. A pilot study of the use of KD in Parkinson’s patients showed a global enhancement in the Unified Parkinson’s Disease Rating Scale scores in all five of its patients, including motor function (Vanitallie et al., 2005). Furthermore, case reports showed improvement in motor functions in patients with Rett syndrome (Haas et al., 1986; Liebhaber et al., 2003) and patients with GLUT1 deficiency (Friedman et al., 2006). Two other studies have used KD in patients with GLUT1 deficiency, mainly to assess short-term effects of its use, including the amelioration of cognitive function. Yet, both of them also highlighted a moderate improvement of motor disorders (Ramm-Pettersen et al., 2014; Bertoli et al., 2015). It should be noted that ketogenic treatments have been studied in patients with Alzheimer’s disease, but mainly for the assessment on the improvement in cognitive functions (Reger et al., 2004; Henderson et al., 2009).

## What Importance Should be Given to KD to Improve Motor Dysfunction?

With respect to the neuroprotective effect of KD involving the different mechanisms previously cited and largely involved in pathophysiological mechanisms of various neurological and neuromuscular diseases, the theoretical benefit of KD is not doubtful. As KD and fasting share similar, potentially beneficial effects, we suspect that fasting could be considered as a therapy. However, this question was raised in a trial evaluating KD versus an intermittent fasting regimen in mice undergoing acute seizure tests (Hartman et al., 2010). Although both strategies were associated with anticonvulsive properties, the mechanisms were different, underlining the need for further studies to fully understand the different strategies.

Contrary to other indications such as epilepsy or cognitive dysfunction, little clinical data is available to assess beneficial effects of KD on motor function. Importantly, KD also has short and long-term, adverse effects regardless of the disease. The classic KD has showed more problems of tolerability than the various modified diets. Major short-term side effects are gastro-intestinal disturbances (which could lead to poor compliance), acidosis, and hypoglycemia (Dhamija et al., 2013; Branco et al., 2016). Long-term side effects include hypercholesterolemia, nephrolithiasis, and premature heart disease (Dhamija et al., 2013). Some authors showed that 15 months of KD in children with intractable epilepsy induced a declining linear growth status with constant weight and resting energy expenditure (Groleau et al., 2014). Clinicians and dietitians currently attempt to prevent KD side effects using gradual initiation of the KD (Bergqvist et al., 2005), alternative diets, such as the modified Atkins diet and the low glycemic index diet, or administering supplements, such as selenium, citrate, calcium, and vitamin D (Bergqvist et al., 2003, 2007; McNally et al., 2009).

The balance between efficacy and toxicity of KD provides substantial promise for such treatment for motor impairment, but it is probably insufficient when administered alone. However, the lack of double-blind, randomized control studies accurately measuring the effects on motor function prevents one from obtaining clear conclusions about this treatment. As some findings about the neuroprotective effect of KD are contradictory, we have to consider the standardization of human and animal protocols. A greater understanding and a better clinical evaluation of KD effects would cause the KD treatment to merit more attention.

## Perspectives of Metabolism-Based Therapeutics in the Management of Neuromuscular Diseases

### Medium-Chain Triglycerides (MCTs)

Medium-chain triglycerides have been studied as an alternative to KD. In the context of ALS, Zhao et al. (2012) reported the recovery of motor function after restoration of energy metabolism in an ALS mouse model treated with MCT (caprylic triglyceride). Although the results were highly promising in enhancing motor performances, there was no effect on animal survival. Nonetheless, this study shows potential for clinical trials. Furthermore, a recent study revealed that triheptanoin, another MCT, protected lumbar motor neurons and delayed onset of motor symptoms in an ALS mouse model (Tefera et al., 2016). Triheptanoin supplementation was also tested in Rett syndrome mice. The results revealed an augmented survival in triheptanoin-fed mice, a gain in social interactions and motor function, an improvement in mitochondrial morphology in skeletal muscle (Park et al., 2014). A diet with MCT was also tested in patients with Alzheimer’s disease, primarily for the evaluation of cognitive function (Ohnuma et al., 2016).

### Deanna Protocol

The Deanna protocol (DP) is a metabolic therapy that provides alternative, energetic fuels. The DP is essentially comprised of arginine alpha-ketoglutarate (AAKG) and other molecules, such as ubiquinol, MCTs, and gamma-aminobutyric acid. The beneficial role of ubiquinol is based on its role in the electron transport chain in mitochondria and ATP production.

Ari et al. (2014) reported that DP would increase motor function and survival in the SOD1-G93A ALS mouse model. ALS mice were fed a SD, KD, or either diets containing ingredients of the DP. SD+DP-fed mice showed a better neurological score than the controls, and KD-fed mice exhibited better motor performance in all motor function tests compared to the controls. The Rotarod test revealed better motor performance in KD mice and the grip test also highlighted better performance in KD and SD+DP mice compared to controls. The Paw Grip Endurance test showed higher motor performance in KD or SD+DP mice than in controls. Significantly, ALS mice fed SD+DP or KD+DP had a remarkable, extended survival time. Authors have suggested that AAKG enhances blood flow, increases muscle protein synthesis, and ameliorates muscle strength though the metabolic role of α-ketoglutarate. MCTs could have a similar function though their conversion to KBs and acetyl-CoA. The production of energetic intermediates bypassing transport and metabolism of glucose, probably deregulated in ALS, may increase motor function in this disease.

### Ketone Esters (KEs)

One therapeutic goal is to replace the KD, and its strict requirements for observance, with dietary supplements that could generate sustained ketosis. KEs are considered to be substitutes for KD and are suitable for oral treatment, compared to KBs. Ingestion of KEs can directly increase blood levels of KBs without the delay observed in KD or fasting (Hashim and VanItallie, 2014). The main KEs used are 1,3-butanediol monoester of βOHB and glyceryl-tri-3-hydroxybutyrate (3GHB) (Brunengraber, 1997). Oral and intravenous administering of KEs demonstrated that they were safe and well-tolerated in animals and humans (Clarke et al., 2012a,b), which makes them an interesting complementary treatment.

Ketone ester supplementation has been shown to relieve symptoms in AS mouse models (Ciarlone et al., 2016). Positive effects of KEs have been observed in Alzheimer’s mouse models with an improvement in energy metabolism and a reduction in amyloid-β deposition (Zhang J. et al., 2013; Pawlosky et al., 2017). A study in one patient with Alzheimer’s disease treated with KEs exhibited a gain in cognitive function and physical activity (Newport et al., 2015).

## Conclusion

The perspective of the use of KD in a variety of diseases has been growing these past recent years. This adjuvant therapy has shown interesting potential in neurological and neuromuscular diseases. The obtained experimental results point out the neuroprotective role of the increase of KBs levels and the reduction of blood glucose, in association with the involvement of various signaling pathways (e.g., IGF-1/AKT/mTOR, AMPK) and effects on inflammation and oxidative status. The utility of KD and its variations for the treatment of neuromuscular diseases suggest a central mechanism in restoring energy metabolism, especially in disease with an impairment of glucose metabolism. Although various studies have suggested a positive effect of KD in a number of neuromuscular diseases, several obstacles remain to be tackled before these findings can be applied widely in the clinic, and further research is necessary to elucidate the mechanisms that mediate the neuroprotective effects. Indeed, several points remain unexplored, as for example the direct effects of KBs on gene expression. Many questions remain unanswered: is there a neuroprotective effect of KD in all conditions, pathological or physiological? If the KD can be beneficial for the brain, can it be deleterious for other organs? How long an exposure to the diet is necessary to confer long-term benefit? Does a diet monitoring improved efficacy? It should also be noted that many studies on KD were performed on animal models, highlighted that clinical trials of its use in affected patients are essential.

The effects of KD involve numerous mechanisms highlighting specific pathways that can represent interesting therapeutic approaches. Despite the well-documented advantage of KD in the treatment of several diseases, the adverse effects should also be acknowledged. More and more studies search for alternative treatments or diets to KD, presenting similar effects with fewer adverse side effects and fewer daily constraints for patients. However, these diets might represent an exceptional option as a co-adjuvant therapy.

## Author Contributions

CV-D and HB wrote the manuscript and designed the review. RH helped write new paragraphs of the manuscript to perform responses to reviewers. CV-D and RH edited the manuscript following reviewers comments. CV-D, PR, VP, RH, PC, CA, and HB have been involved in drafting the manuscript or revising it critically for important intellectual content. All authors contributed to the conception of this review article. All authors read and approved the final manuscript.

## Conflict of Interest Statement

The authors declare that the research was conducted in the absence of any commercial or financial relationships that could be construed as a potential conflict of interest. The reviewer YN and handling Editor declared their shared affiliation.

## Abbreviations

ALS: amyotrophic lateral sclerosis
AS: Angelman syndrome
ATP: adenosine triphosphate
βOHB: β-hydroxybutyrate
DP: Deanna protocol
GABA: gamma amino-butyric acid
KBs: ketone bodies
KD: ketogenic diet
KEs: ketone esters
LCTs: long-chain triglycerides
MCTs: medium-chain triglycerides
MCT: monocarboxylate transporter
NADH: nicotinamide adenine dinucleotide
ROS: reactive oxygen species
SD: standard diet
TCA: tricarboxylic acid
